# Supplementary figures and images for: Combining CD3/GD2 bispecific T cell engager with human Vγ9Vδ2 T cells facilitates neuroblastoma cell targeting and killing in vitro
Source: PLoS One. 2025 Jun 9;20(6):e0325389. doi: 10.1371/journal.pone.0325389 (PMC12148185; doi:10.1371/journal.pone.0325389)

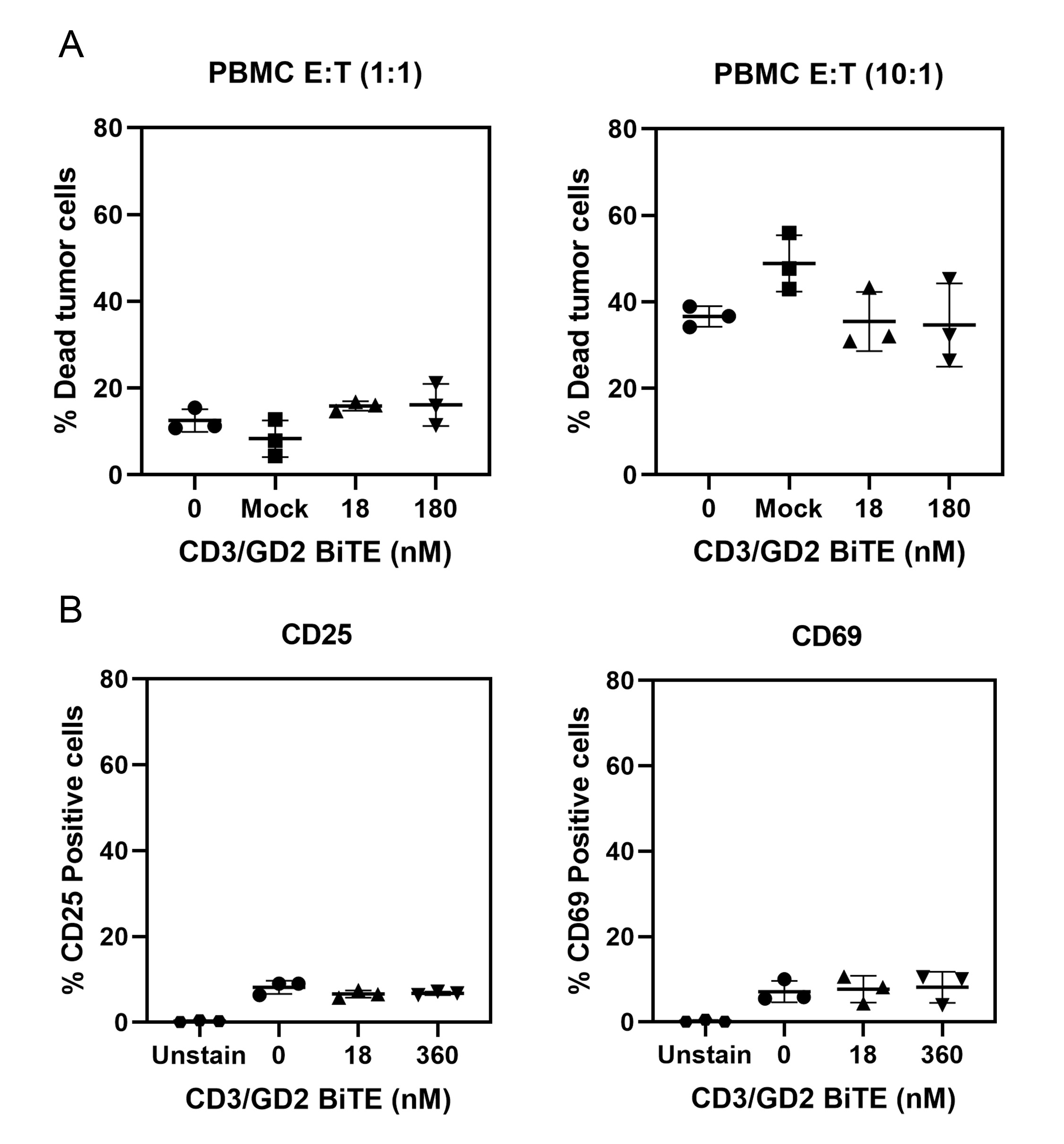

Supplement: S1 Fig — (A) The mean percentage of dead tumor cells from PBMC armed with CD3/GD2 BiTE at E:T ratios of 1:1 and 10:1. Human PBMCs were primed with CD3/GD2 BiTE at concentrations of 18 and 180 nM before being added to CFSE-stained SH-SY5Y cells for 24 h. Dead cells were stained with 7-AAD. Dead tumor cells positive for both CFSE and 7-AAD were detected and measured using flow cytometer. (B) The expression of activation markers CD25 and CD69 on PBMCs after co-culture with CD3/GD2 BiTE. The PBMCs were co-cultured with CD3/GD2 BiTE at concentrations of 18 and 360 nM for 24 h. PBMCs were then stained with anti-CD25 or anti-CD69 antibodies and analyzed by flow cytometry. The dot plots were generated from three independent experiments, and the results are expressed as means ± SD. (TIF) [file pone.0325389.s001.tif]
