# Supplementary material for: Combining CD3/GD2 bispecific T cell engager with human Vγ9Vδ2 T cells facilitates neuroblastoma cell targeting and killing in vitro
Source: PLoS One. 2025 Jun 9;20(6):e0325389. doi: 10.1371/journal.pone.0325389 (PMC12148185; doi:10.1371/journal.pone.0325389)
Supplement: S1 File — (ZIP) [file pone.0325389.s002.zip › CD3-GD2 BiTE Raw data/CD3-GD2 BiTE Uncropped gels.docx]

**Uncropped gels**

**Figure 1B**: Periplasmic and cytoplasmic proteins of human scFv CD3 clones.

Western blot analysis using anti-E tag antibody.

**Lanes (left to right)**

| Land M | Protein Ladder |
| --- | --- |
| Lane 1 | Periplasmic protein of *E. coli* HB2151 |
| Lane 2 | Periplasmic protein of *E. coli* HB5121 containing human scFv CD3 clone 6 |
| Lane 3 | Periplasmic protein of *E. coli* HB5121 containing human scFv CD3 clone 18 |
| Lane 4 | Periplasmic protein of *E. coli* HB5121 containing human scFv CD3 clone 85 |
| Lane 5 | Cytoplasmic protein of *E. coli* HB2151 |
| Lane 6 | Cytoplasmic protein of *E. coli* HB2151 containing human scFv CD3 clone 6 |
| Lane 7 | Cytoplasmic protein of *E. coli* HB2151 containing human scFv CD3 clone 18 |
| Lane 8 | Cytoplasmic protein of *E. coli* HB2151 containing human scFv CD3 clone 85 |

**M 1 2 3 4 5 6 7 8**


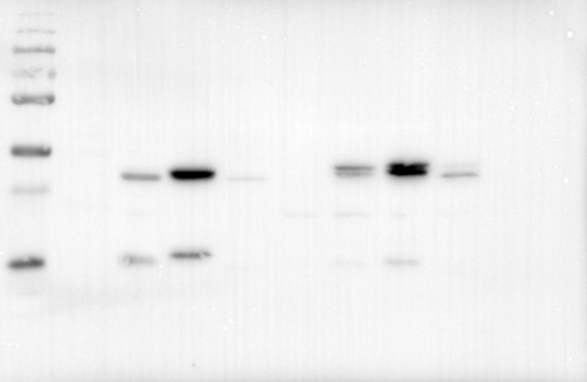


**Figure 2C**: Reducing-PAGE analysis of purified CD3/GD2 BiTE protein stained with InstantBlue^TM^.

CD3/GD2 BiTE was produced by transfection for 72 h. After transfection, the culture medium was collected to purify the bispecific antibody protein using Ni-NTA agarose. The purified CD3/GD2 BiTE protein was further characterized by SDS–PAGE and Western blot analysis.

**Lanes (left to right)**

| Lane 1 | Culture medium before purification |
| --- | --- |
| Lane M | Protein Ladder |
| Lane 2 | Culture medium flow through after purification |
| Lane 3 | Wash flow through after purification |
| Lane 4 | Purified CD3/GD2 BiTE protein after purification |

**1 M 2 3 4**


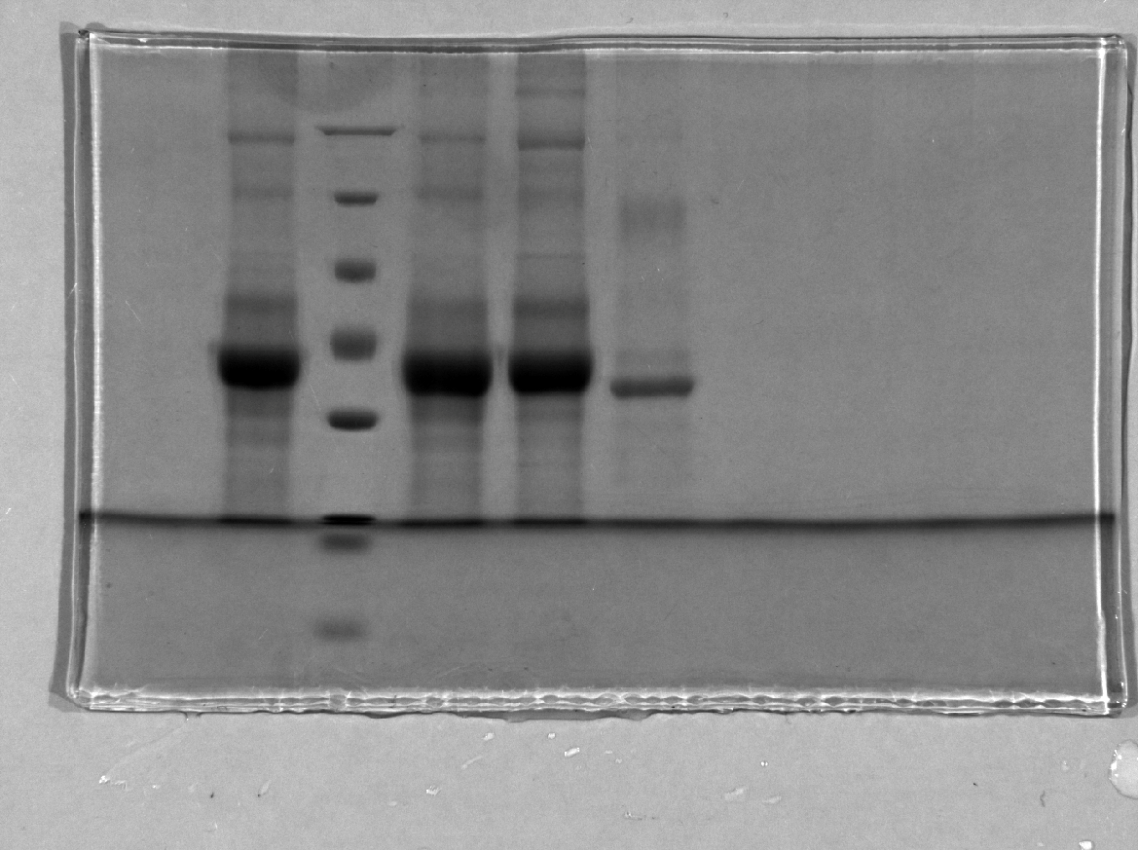


**Figure 2C**: Western blot analysis of purified CD3/GD2 BiTE detected using an anti-histidine tag.

**Lanes (left to right)**

| Lane M | Protein Ladder |
| --- | --- |
| Lane 1 | Culture medium flow through after purification |
| Lane 2 | Wash flow through after purification |
| Lane 3 | Elute fraction after purification |
| Lane 4 | Purified CD3/GD2 BiTE concentrated by Amicron ® Ultra Centrifugation Filter, 30 kDa MWCO |
| Lane 5 | Purified CD3/GD2 BiTE concentrated by Amicron ® Ultra Centrifugation Filter, 10 kDa MWCO |

**M 1 2 3 4 5**


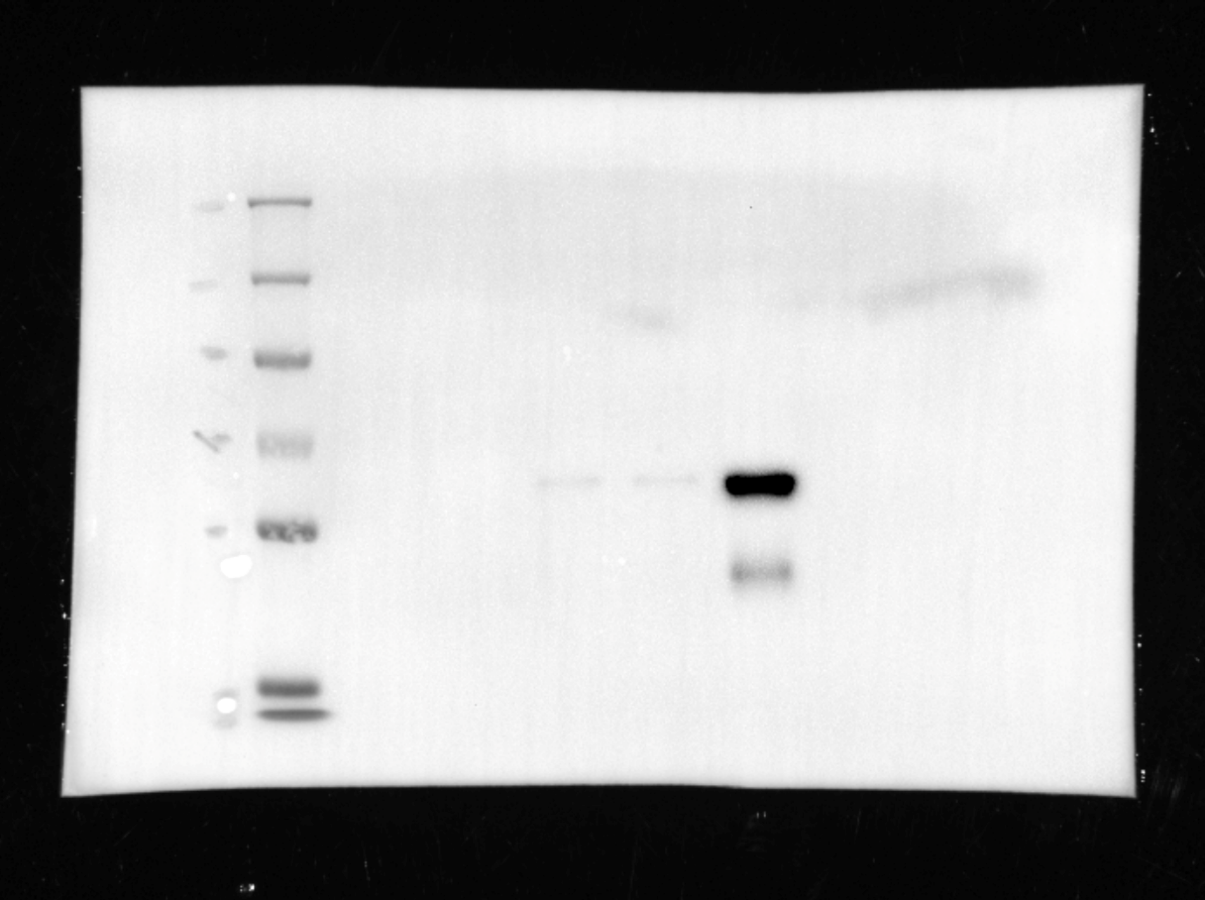


**Figure 2D**: Non-reducing-PAGE analysis of CD3/GD2 BiTE treated with DTT at different concentrations, stained with InstantBlue^TM^.

CD3/GD2 BiTE protein were incubated with the reducing agent DTT at various concentrations at 37°C for 30 minutes. The reduced protein was then subjected to 12% SDS-PAGE under native conditions and stained with InstantBlue^TM^.

| Lane M | Protein Ladder |
| --- | --- |
| Lane 1 | 10 µg of CD3/GD2 BiTE without DTT treatment |
| Lane 2 | 10 µg of CD3/GD2 BiTE treated with 1 mM DTT |
| Lane 3 | 10 µg of CD3/GD2 BiTE treated with 10 mM DTT |
| Lane 4 | 10 µg of CD3/GD2 BiTE treated with 50 mM DTT |
| Lane M | Protein Ladder |
| Lane 5 | 10 µg of BSA without DTT treatment |
| Lane 6 | 10 µg of BSA treated with 1 mM DTT |
| Lane 7 | 10 µg of BSA treated with 10 mM DTT |
| Lane 8 | 10 µg of BSA treated with 50 mM DTT |


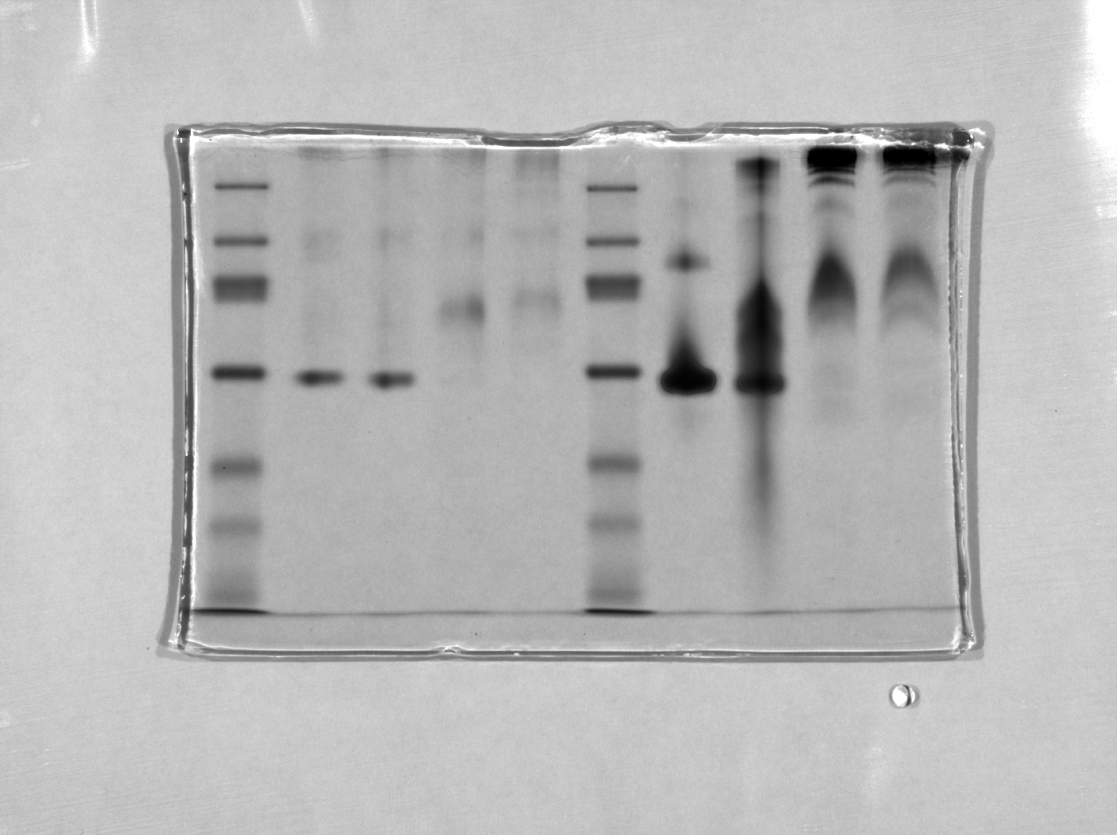


**M 1 2 3 4 M 5 6 7 8**

**Figure 5A**: Fluorescence images of 3D tumor spheroids treated with CD3/GD2 BiTE-armed activated Vγ9Vδ2 T cells.

| CD3/GD2 BiTE | Optical | GFP | EthD-1 | Merge |
| --- | --- | --- | --- | --- |
| 0 nM | 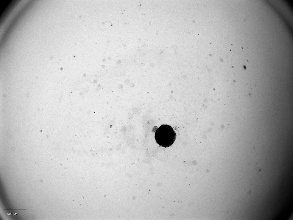 | 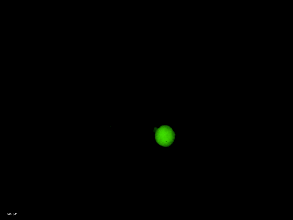 | 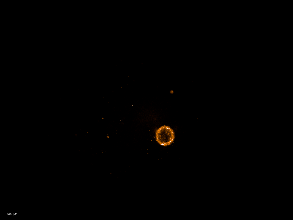 | 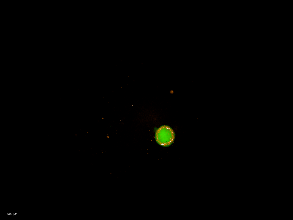 |
| Mock | 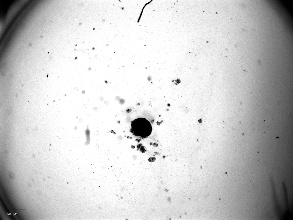 | 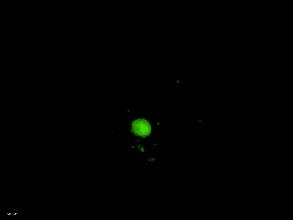 | 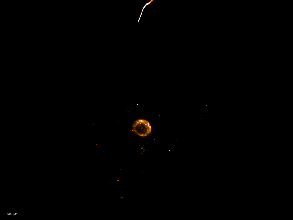 | 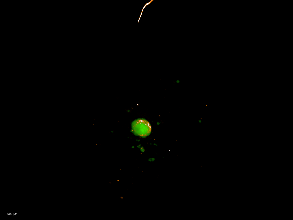 |
| 45 nM | 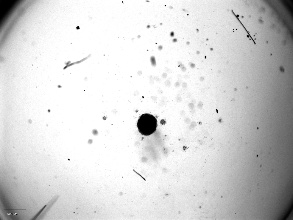 | 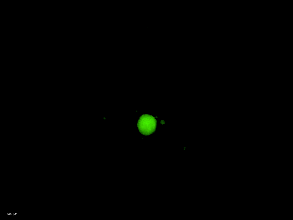 | 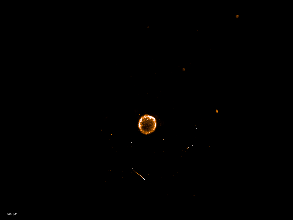 | 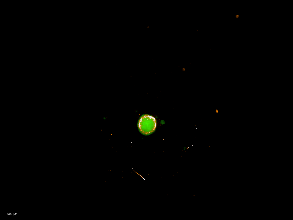 |
| 90 nM | 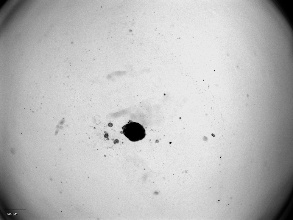 | 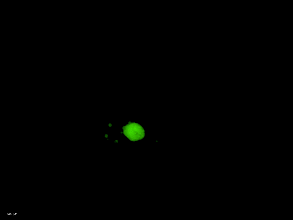 | 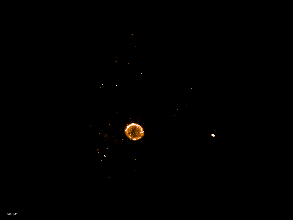 | 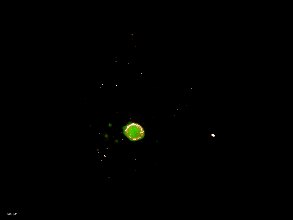 |
| 180 nM | 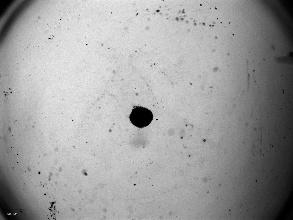 | 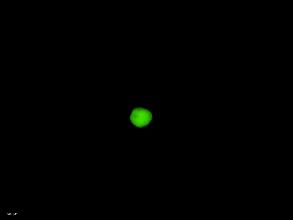 | 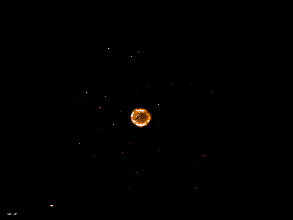 | 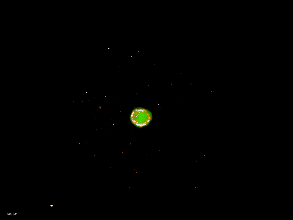 |
| 360 nM | 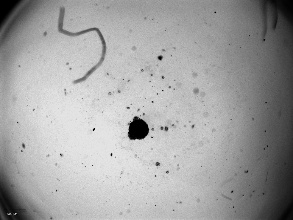 | 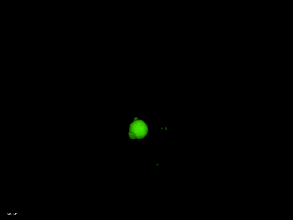 | 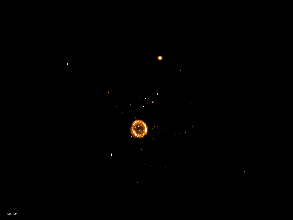 | 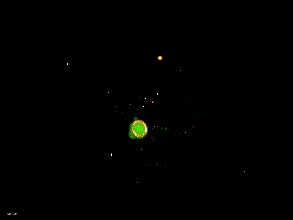 |
