# Supplementary material for: Combining CD3/GD2 bispecific T cell engager with human Vγ9Vδ2 T cells facilitates neuroblastoma cell targeting and killing in vitro
Source: PLoS One. 2025 Jun 9;20(6):e0325389. doi: 10.1371/journal.pone.0325389 (PMC12148185; doi:10.1371/journal.pone.0325389)
Supplement: Attachment — Submitted filename: Response to Reviewers.docx [file pone.0325389.s004.docx]

Department of Pediatrics, Faculty of Medicine Ramathibodi Hospital,

Mahidol University, Bangkok 10400, Thailand

_____________________________________________________________________________________

January 17, 2025

Manuscript Number: **PONE-D-24-27594**

Title: **Combining CD3/GD2 bispecific T cell engager with human Vγ9Vδ2 T cells facilitates**

**neuroblastoma cell killing**

**Dear reviewers,**

We would like to thank you for sending comments following the peer review of our manuscript.

We are very appreciating the interest that reviewers have taken in our manuscript. We would like to gratefully thank the reviewers for providing valuable comments which helped to improve our manuscript markedly. In the revised version, we have addressed concern of the reviewers. Changes to the text are highlighted in yellow.

According to the reviewer’s suggestions, we have modified the title to be “**Combining CD3/GD2 bispecific T cell engager with human Vγ9Vδ2 T cells facilitates** **neuroblastoma cell targeting and killing *in vitro”***

We do hope that the revised version of our study is now suitable to publish in PLOS ONE.

Thank you for your consideration of our revised manuscript.

Yours sincerely,

Suradej Hongeng, MD

**Reviewer #1:**

**Major comments**

In the present manuscript, the authors present the construction of bispecific T-cell engager composed of an anti-GD2 and an anti-CD3 targeting unit, to activate the gamma/delta T cells for killing in response to a CD2-positive neuroblastoma cell line. They expressed the molecule at a high concentration in HEK cells, purified it with a Ni-NTA-based affinity chromatography, and tested it for the activity. The latter included binding to both types of antigen-positive cell lines (effector and target), and the killing activity for the target cells. The BiTE activity is also examined in a 3D culture of tumor cells (as a matrix-embedded cell line), where armed gamma/delta T cells also show killing capability as judged by fluorescent microscopy.

The concept of the study is very valuable, as (1) the effector cell group used here, the gamma/delta T cells, emerged in the last decades as a powerful mechanism of tumor combat, and (2) the neuroblastoma is a condition where novel therapy concepts are certainly required. However, there is a number of questions to be addressed:

1. Discovery of anti-CD3 molecule: all data on discovery and characterization are missing, and how was it made sure that the chosen clone is not an agonist to CD3? Outcomes to procedures described in lines 155-159: the results of anti-CD3 epsilon screening with sequencing of relevant clones, and the results of the activity of the selected CD3 clones (basic biophysical characterization with SDS-PAGE, activity with ELISA, including potential cross-reactivity with unrelated antigens, and sequencing for identity). How was the decision on using this particular anti-CD3 antibody based, and in particular, how was the agonism excluded?

Reply: Thank you for your valuable suggestion. In this study, we initially isolated human scFv against CD3 epsilon from an M13 phage library displaying human scFv fragments using phage panning technology with recombinant CD3 epsilon. After phage panning, we screened 90 clones for human scFv gene insert by colony PCR. Sixty clones containing human scFv gene insert were subjected to induce protein expression and the total cel lysate of each clone was evaluated by indirect ELISA. Human scFv CD3 clones 18, 6 and 85 demonstrated the highest signals in the indirect ELISA, respectively (Fig 1A). To confirm, human scFv CD3 clones 18, 6 and 85 were selected for further expression in higher culture volume and extraction for the periplasmic and cytoplasmic protein. The fractionated proteins were analyzed by western blot analysis and indirect ELISA. We observed the protein bands, with an approximately size of 28 kDa from all human scFv CD3 clones (Fig 1B). Notably. human scFv CD3 clone 18 markedly demonstrated the highest binding signal from both periplasmic and cytoplasmic fractions by indirect ELISA (Fig 1C). Bovine serum albumin was used as an irrelevant antigen in indirect ELISA to ensure the non-specific binding. The absorbance of soluble human scFv CD3 against CD3 epsilon was analyzed by subtracting the absorbance of the negative protein in CD3 epsilon, followed by the absorbance of soluble human scFv CD3 in BSA-coated well, respectively. The amino acid sequence of human scFv CD3 is shown in Fig 1D. The CDRs on the VH and VL regions, and flexible linker are indicated by colored and underlined text. Overall, human scFv CD3 clone 18 was selected to construct CD3/GD2 BiTE.

In the revised manuscript, we have included details about the discovery of human scFv CD3 in the method (Page 9 lines 185-198 and Page 10 lines 199-209), result (Page 15 lines 331-342) and Page 16 lines 343-352), Fig 1 and discussion section (Page 20 line 463 and Page 21 lines 464-467).

2. Characterization of the chosen BiTE molecule: for validation of binding data, a size-exclusion chromatography profile showing that the studied molecule is a monomer, must be shown.

Reply: Due to the limited resources for performing size exclusion chromatography, we instead investigated the protein dimerization of CD3/GD2 BiTE under reducing conditions. CD3/GD2 BiTE was treated with a reducing agent, DTT, before running in non-reducing PAGE. The result is shown in Fig 2D. When CD3/GD2 BiTE was dissociated into the monomer subunit by incubating with 1 mM DTT, we observed one single band located at the same size of CD3/GD2 BiTE without DTT. This confirmed that CD3/GD2 BiTE protein folds as a monomeric protein.

We have included the protein dimerization investigation in the revised version as highlighted. Method (Page 13 lines 282-287), Result (Page 17 lines 368-373), Discussion (Page 21 lines 476-488)

3. Conceptual: why is this BiTE expected to engage with gamma/delta T cells, if CD3 is present also on several other T cells? How does this BiTE differentiate from other BiTEs in this aspect? A control anti-CD3 molecule would be very valuable for all experiments.

Reply: Initially, we would like to isolate the human scFv that bind to CD3 molecule and also activate T cells to promote cytotoxicity. Unlike other anti-CD3 antibody, our human scFv CD3 clone 18 could recognize the CD3 molecule but not activate T cells or mediate cytotoxicity. Therefore, suitable effector cells for CD3/GD2 BiTE need to mediate the cytotoxicity themselves. The γδ T cell is an interesting T cell population used in cancer immunotherapy since this type of T cell can be expanded and activated *ex vivo.* The activated γδ T cells themselves have ability to kill broad spectrum of tumors and malignancies. Therefore, in this study, we aimed to use CD3/GD2 BiTE to improve the binding specificity and avidity of activated Vγ9Vδ2 T cells against neuroblastoma.

We have stated this in the abstract (Page 2 lines 23-25) introduction (Page 7 lines 137-138) and discussion (Page 25 lines 571-573).

4. Methodology: it must be shown that the anti-CD3 clone is specific to CD3 (by using a CD3-negative T-cell line for experimental control), and that the BiTE is not active on GD2-negative cells.

Reply: We have included the GD2-negative cell line, SK-N-SH cells, and CD3-negative cell line, Raji cells, for evaluating the binding specificity of CD3/GD2 BiTE by flow cytometry. As shown in Fig 3, CD3/GD2 BiTE exhibited binding reactivity to GD2-positive SH-SY5Y cells and CD3-positive Jurkat cells in dose-dependent manner. Whereas the percentage of positive cells for GD2-negative SK-N-SH cells and CD3-negative Raji cells found below 10% at all tested concentrations of CD3/GD2 BiTE. This indicated the specificity of CD3/GD3 BiTE to its target antigens, GD2-positive SH-SY5Y cells and CD3-positive Jurkat cells.

We have included this in the method (Page 13 lines 290-292) and result (Page 17 lines 378-380).

5. Results and outlook: the molecule (if negative controls which are to be delivered indeed confirm the data presented) is not very potent as over 100 nM concentrations are required for the effects. Is this due to low affinity towards any or both of the antigens? Which measures will be taken to achieve a more potent activation of T cells?

Reply: Since CD3/GD2 BiTE demonstrated only binding reactivity to T cell but did not activate T cell function, the objective of combining CD3/GD2 BiTE with activated Vγ9Vδ2 T cells is to enhance the binding specificity and avidity against neuroblastoma cells. Therefore, the concentration of CD3/GD2 BiTE used in the cytotoxic assay was higher than that of other BiTEs which can bind and activate T cell function 10 to 20 times (Ganesan R. et al., 2021, Fei K. et al., 2022 and Yankelevich M. et al., 2012). Even though CD3/GD2 BiTE itself could not activated T cell function but it could enhance tumor cell killing with 5-10 times fewer effector cells when using Vγ9Vδ2 T cells.

We have stated this in the introduction (Page 7 lines 137-138) and discussion (Page 25 lines 571-573).

6. Lines 136-139: the results of monitoring of gamma/delta T cells characterization and activation?

Reply: In the revised manuscript, we have included the methods and results of characterization of human Vγ9Vδ2 T lymphocytes by multiparametric immunophenotyping. Highlighted in Page 9 lines 175-184 and Page 18 lines 392-402.

**Minor comments**

Please find below a list of remarks which I hope will be helpful.

7. Lines 8-9: „We created a bispecific T cell engagers antibody against CD3 and disialoganglioside (GD2), called CD3/GD2 BiTEs“ – a very particular use of singular and plural, please correct

Reply: We have changed all the plural words (CD3/GD2 BiTEs) to be a singular word (CD3/GD2 BiTE) throughout the text.

8. Line 60: “is currently being revolutionized“ – remodelled would be a better expression; one part of the molecule is kept constant while the TAA-targeting part is changed for another specificity

Reply: We have changed the word as recommended in Page 5 line 100.

9. Line 63: CD3 molecule

Reply: We have changed the word as recommended in Page 6 line 102.

10. Lines 64-66: this sentence is not ok and needs to be re-written

Reply: We have re-written the sentence “however severe side effects by stimulating pan-T cells including various immunoregulatory and immunosuppressive T cells leading to immune evasion and cytokine storm need to be considered” to be the sentence below in Page 6 lines 102-107.

“Recently, the safety and efficacy of BiTEs targeting CD3 molecules and TAAs have been explored in more than 100 clinical trials. However, several key challenges remain with BiTEs, including the recruitment of counterproductive CD3+ T-cell subsets, the release of systemic cytokines, the expansion of immune checkpoint molecules, the presence of an immunosuppressive tumor microenvironment, tumor antigen loss or escape, on-target off-tumor toxicity, and suboptimal potency.”

11. Line 71: “GD2 can be considered as a tumor associated antigen which is an important target for the treatment of NB”: please reword: GD2 can be considered as a tumor associated antigen and important target for the treatment of NB.

Reply: We have changed the word as recommended in Page 5 line 77.

12. Line 78: “Despite, monoclonal anti-GD2 antibody has potentially suitable” – Despite the potential suitability of the monoclonal anti-GD2 antibody

Reply: We have changed the word as recommended in Page 5 lines 83-84.

13. Line 84: “The predominant type of γδ cells in human peripheral blood”

Reply: We have changed the word as recommended in Page 6 lines 110-111.

14. Line 90: “the safety of Vγ9Vδ2 T cells-based immunotherapy in hematologic malignancies has been reported“ – the safety issues with … have been reported

Reply: We have changed the word as recommended in Page 6 lines 116-117.

15. Line 93: “for more than three decades” – since more than three decades

Reply: We have changed the word as recommended in Page 6 line 119.

16. Line 101: bispecific T-cell engager antibody, and please correct throughout the text

Reply: We have corrected throughout the revised manuscript.

17. Line 106: “on neuroblastoma cell line and 3-dimentional culture” – and in 3-dimensional culture

Reply: We have corrected throughout the revised manuscript.

18. Line 115: “T cell leukemia cell lines, Jurkat were” – T-cell leukemia cell lines and Jurkat cells

Reply: We have corrected as recommended in Page 8 lines 150-151.

“Jurkat cells, a T-cell leukemia cell line, and Raji cells, a B-cell lymphoma line…….”

19. Lines 118-119: “were incubated at 37˚C, 5% CO2 and 95% humidified atmosphere”: CO2, 2 in subscript, and please check the percentage of humidification (usually it is around 80)

Reply: We have corrected as recommended in Page 8 lines 155 to be “70-80% humidified atmosphere”

20. Line 132: “RPMI 11640 medium” – 1640

Reply: We have corrected as recommended in Page 8 lines 168 to be “RPMI 1640”.

21. Line 137: “cells were characterized the cell surface phenotype” – for the cell surface phenotype

Reply: We have extended the method for characterization of human Vγ9Vδ2 T lymphocytes by multiparametric immunophenotyping in Page 9 lines 175-184.

“**Characterization of human Vγ9Vδ2 T lymphocytes by multiparametric immunophenotyping**

The expression of cell surface markers on activated Vγ9Vδ2 T lymphocytes was analyzed using flow cytometric antibody staining. Prior to staining, an Fc block solution (BioLegend, USA) was added to the cells to prevent non-specific Fc receptor binding. 7-Aminoactinomycin D (7-AAD) (eBioscience, CA, USA) was used to differentiate live cells from dead cells. Antibodies specific for CD45-Pacific Blue (Clone: 2D1), CD3-APC (clone: OKT3), Vγ9-FITC (Clone: B3), and Vδ2-PE (Clone: B3) were obtained from BioLegend (San Gabriel, CA, USA). Multiparameter flow cytometric analysis was performed using a FACSLyric flow cytometer (BD Biosciences, USA).”

22. Line 138, and throughout the manuscript: antibodies should be cited with RRIDs (Antibody Registry, <https://www.antibodyregistry.org/faq>)

Reply: Thank you very much for this recommendation. We found that some antibodies in the revised manuscript were not registered into the antibody database. We would like to keep this cited antibody format throughout our revised manuscript.

23. Line 150: how many wells were used for panning? And “Unbound phages were removed by the washing buffer 10 times”

Reply: We have added the information as recommended below.

“Recombinant CD3 epsilon, at a concentration of 0.5 µg, was coated onto 8 ELISA wells” in Page 9 line 190.

“Unbound phages were removed by washing with 300 µl of washing buffer 10 times.” In Page 9 lines 195-196.

24. Line 151: “E. coli strain HB2151 was added into the well” – the volume of the exponential culture per well and in total? Volume for E.coli

Reply: We have added the information as recommended below.

“Afterward, 200 µl of log-phase E. coli strain HB2151..” in Page 9 line 198.

25. Line 156: “tested the binding activity of soluble human” – tested for the binding activity

Reply: We have changed as recommended. Page 10 line 205.

26. Line 157:” HRP conjugated anti-E tag” – please specify the reagent company, country

Reply: We have added the information as recommended below.

“HRP-conjugated anti-E tag (GE Healthcare, UK).” Page 10 lines 206-207.

27. Line 178: “CD3/GD2 BiTEs protein at 2-10 μg was performed SDS-polyacrylamide gel
    electrophoresis“ – surely, the analysis of the protein was performed

Reply: We have changed as recommended. Page 12 line 263.

“Protein analysis was conducted….”

28. Line 180: “On the other hand,” – additionally is a better expression

Reply: We have changed as recommended. Page 12 line 266.

29. Line 259: “measure the number of CD3/GD2 BiTEs bound on the target cells“ – the number is not really determined here, so the binding activity or similar would be a better expression

Reply: We have changed to “to measure the percentage of positive cells.” Page 17 line 382.

30. Line 264: was elevated

Reply: We have changed as recommended. Page 17 line 385.

31. Line 265: “exhibited similar characterizations” – appeared similar

Reply: We have changed as recommended. Page 17 line 386.

32. Line 267: “Consequently, the CD3/GD2 BiTEs demonstrated” – To conclude, the CD3/GD2…

Reply: We have changed as recommended. Page 17 line 389.

33. Line 297: nM (nanomolar), where the percentage

Reply: We have changed the sentence to be “Significant cytotoxic effects were observed at CD3/GD2 BiTE concentrations of 180 and 360 nM, with 38.10±4.20% and 42.30±4.45% dead tumor cells, respectively. Page 19 line 422-423.

34. Line 299: word order: “enhanced 1.57-fold and 1.75-fold higher cytotoxicity compared” – enhanced the cytotoxicity 1.57-fold and 1.75-fold

Reply: We have changed as recommended. Page 19 lines 424-425.

35. Line 300: “Mock control demonstrated the similar dead tumor cell percentage of non-treated CD3/GD2 BiTEs“ – this is an important statement and should not be affected by poor grammatic structure – in mock control, the percentage of dead tumor cells was similar to when the cells were not treated with CD3/GD2 BiTEs- does it hold true for all E:T ratios?

Reply: We have changed as recommended.

“The mock control showed no significant differences across all E:T ratios, without CD3/GD2 BiTE treatment.” Page 19 lines 425-426

36. Lines 304-306: important result, again not easy to understand: “using CD3/GD3 BiTEs to increase the specificity of activated Vγ9Vδ2 T cells against neuroblastoma cells reduced five to tentimes in the number of effector cells” – the addition of the CD3/GD2 BiTEs enabled tumor cell killing at 5-10-lower number of effector cells.

Reply: We have changed the sentence as recommended.

“Notably, cytotoxicity of CD3/GD2 BiTE at 360 nM with an E:T ratio of 1:1 (Fig 4C) was comparable to that of activated Vγ9Vδ2 T cells alone at E:T ratios of 5:1 and 10:1 (Fig 4B), demonstrating that CD3/GD2 BiTE enhanced tumor cell killing with 5-10 times fewer effector cells.” Page 19 lines 427-430.

37. Line 315: “with non-treated CD3/GD2 BiTEs” – with the outcomes of the cell systems without BiTe addition.

Reply: We have changed as recommended.

“in comparison to the outcomes of the cell systems without BiTE addition.” Page 19 line 439 and Page 20 line 440.

38. Lines 317-318: “The CTCF fold change of lived SH-SY5Y GFP cells was significantly observed“ – the change in the number of live target cells was significant

Reply: We have changed as recommended.

“The reduction in the number of live target cells was significant, at 360 nM of CD3/GD2 BiTE (p < 0.05).” Page 20 lines 442-443.

39. Line 318: “(p < 0.05). While the signal of EthD-1“ – please make one sentence

Reply: We have changed as recommended.

“Meanwhile, the EthD-1 signal, indicating dead cells, significantly increased in a dose-dependent manner, ranging from a 1.75 to 2.20-fold CTCF change.” Page 20 lines 443-445.

40. Line 331: human anti-mouse antibody (HAMA) response

Reply: We have changed as recommended. Page 20 line 459.

41. Line 339:” built by joining a single polypeptide chain by flexible linker” – built by joining 2 single-chain antibody fragment sequences

Reply: We have changed as recommended. Page 21 line 469.

42. Line 341: “bispecific bivalent scFv platform” – the fragment described is monovalent for each of its specificities, and as it is only one, it is not a platform.

Reply: We have changed as recommended. Page 21 line 471.

43. Line 344:” which employe the production” – employed for production

Reply: We have changed as recommended. Page 21 line 475.

44. Lines 351-352:” appropriate folding, human-like glycosylation, and post-translation modifications“ – post-translational modifications, and these data would be very enriching for the manuscript. Is the glycosylation or post-translational modifications relevant – as the sequences are not given in the manuscript, it is difficult to judge on this point.

Reply: We agreed with the reviewer. Therefore, we have changed the sentence to be “The use of mammalian expression system enabled proper folding and post-translation modifications, resulting in high productivity and biological activity. Page 21 line 486-488.

45. Lines 358-359: “BiTEs exhibited only recognition ability to their targets without any effect on T cell activation and cytotoxicity against neuroblastoma cell line (data not shown)“ – indeed, these data should be shown because here novel reagents are described. As mentioned above, T-cell activation without a target, as well as the cytotoxic effect without the relevant target cells, must be investigated.

Reply: We have provided the result (S1 Fig.) and discussion of *in vitro* cytotoxicity assay using PBMCs as effector cells in Page 22 line 513 and Page 23 lines 514-526.

“We conducted an *in vitro* cytotoxicity assay using PBMCs as effector cells. PBMCs were primed with CD3/GD2 BiTE at concentrations of 18 and 180 nM before being added to CSCF-stained SH-SY5Y cells at E:T ratios of 1:1 and 10:1 for 24 h. The percentage of dead tumor cells in PBMCs armed with CD3/GD2 BiTE did not significantly differ from PBMCs without CD3/GD2 BiTE or the mock control (S1A Fig.). To further evaluate BiTE-mediated T cell activation, PBMCs were co-cultured with CD3/GD2 BiTE at concentrations of 18 and 360 nM for 24 h. Subsequently, PBMCs were stained with antibodies specific to T cell activation markers CD25 and CD69. The percentage of CD25+ and CD69+ cells in PBMCs co-cultured with CD3/GD2 BiTE was less than 10%, which was comparable to PBMCs without BiTE treatment (S1B Fig.). These results indicate that CD3/GD2 BiTE only recognized the CD3 molecule but did not activate T cells or mediate cytotoxicity against neuroblastoma cell lines. Thus, PBMCs were not suitable effector cells for CD3/GD2 BiTE.”

46. Line 359- 360 “…Although the cytotoxic function of BiTEs by redirecting T cells via CD3 is attractive. There are“ – please make one sentence out of these two

Reply: Thank you for kindly recommending. We have removed this paragraph from the discussion section.

47. Line 361: effect on the immunoregulatory and...

Reply: Thank you for kindly recommending. We have removed this paragraph from the discussion section.

48. Line 368: “administration of natural and bispecific antibodies” – administration of T-cell specific natural and …

Reply: Thank you for kindly recommending. We have removed this paragraph from the discussion section.

49. Lines 370-371: “without activation of effector cells“ - without unspecific activation of effector cells

Reply: Thank you for kindly recommending. We have removed this paragraph from the discussion section.

50. Lines 382-383: word order: “combined CD3/GD2 BiTEs with activated Vγ9Vδ2 T cells“ - CD3/GD2 BiTEs combined with activated Vγ9Vδ2 T cells

Reply: We have changed as recommended. Page 23 lines 537-538.

51. Line 386: nM (nanomolar)

Reply: We have corrected throughout the revised manuscript.

52. Line 391: „to non-treated CD3/GD2 BiTEs” – to tumor cells incubated with effector cells without the presence of CD3/GD2 BiTEs

Reply: We have changed the phrase to be “with effector cells alone”. Page 24 line 542.

53. Line 399: which results in different phenotypes and effector cell characteristics

Reply: We have changed the phrase to be “which can result in diverse phenotypes and effector characteristics” Page 24 line 549.

54. Line 409: in a format of a CD3/GD2 BiTEs

Reply: We have changed as recommended. Page 24 line 562.

55. Line 411: retained the binding reactivity

Reply: We have changed as recommended. Page 24 line 566.

56. Line 415: proposes an alternative strategy – it is not provided quite yet

Reply: We have changed as recommended. Page 24 line 577.

57. Figure 2. Missing the titration of tumor and effector target-negative cells.

Reply: We have already added the result of binding activity of CD3/GD2 BiTE on CD3-negative Raji cells and GD2-negative SK-N-SH cells as shown Fig 3.

58. Figure 3. Can you explain why higher concentrations of BiTE were used along with higher numbers of effector cells?

Reply: CD3/GD2 BiTE only recognized the CD3 molecule but did not activate T cells or mediate cytotoxicity. In addition, CD3/GD2 BiTE improved the tumor specificity and avidity of Vγ9Vδ2 T cells against neuroblastoma cells. However, the concentration of BiTE was used depended on the number of effector cells in each E:T ratio. To compare the cytotoxic ability of combined CD3/GD2 BiTE and Vγ9Vδ2 T cells at different E:T ratio, we selected the concentrations of CD3/GD2 BiTE that armed Vγ9Vδ2 T cells up to 50% and 100% positive cells.

**Reviewer #2:**

The authors present a potentially promising approach to the treatment of recurrent or relapsed neuroblastoma. As others have shown CD3+ T cells, including gamma delta (gd) T cells can be directed to kill tumors cells through engagement of bispecific T cell engagers. The engager used in the present study targets CD3 on T cells and GD2 on neuroblastoma cells. GD2 is the target of a commercial monoclonal antibody, dinutuximab. Overall, the materials used and studies presented are well designed, characterized and analyzed. However, several concerns remain as stated below:

1. It would benefit the study to demonstrate the effectiveness of the approach against multiple neuroblastoma cell lines.

Reply: Thank you very for your suggestion. In the revised manuscript, we have included the GD2-negative cell line, SK-N-SH cells, and CD3-negative cell line, Raji cells, for evaluating the binding specificity of CD3/GD2 BiTE using flow cytometry. As shown in Fig 3, CD3/GD2 BiTE exhibited binding reactivity to GD2-positive SH-SY5Y cells and CD3-positive Jurkat cells in dose-dependent manner. Whereas the percentage of positive cells for GD2-negative SK-N-SH cells and CD3-negative Raji cells found below 10% at all tested concentrations of CD3/GD2 BiTE. This indicated the specificity of CD3/GD3 BiTE to its target antigens. (Page 17 lines 378-380)

According to the negative binding activity of CD3/GD2 BiTE on GD2-negative SK-N-SH cells, we did not perform cytotoxicity against GD2-negative SK-N-SH cells. In this experiment, we selected GD2-positive SH-SY5y cells to be the tumor target cells since SH-SY5Y demonstrates high level expression of GD2 molecule on the cell surface. Activated Vγ9Vδ2 T cell alone without CD3/GD2 BiTE treatment and mock control using irrelevant protein were used to ensure the ability of CD3/GD2 BiTE to target neuroblastoma cell.

2. Demonstration of in vivo activity in a neuroblastoma tumor model would further benefit the study.

Reply: We agree with the reviewer that *in vivo* cytotoxicity would be performed to confirm the ability and safety of CD3/GD2 BiTE. CD3/GD2 BiTE only recognized the CD3 molecule but did not activate T cells or mediate cytotoxicity. However, CD3/GD2 BiTE improved the tumor specificity and avidity of Vγ9Vδ2 T cells against neuroblastoma cells. In this study, we proposed the concept for enhancing neuroblastoma cell killing by priming the activated effector cells with the binding antibody. We test the ability of CD3/GD2 BiTE to effectively redirected Vγ9Vδ2 T cells against neuroblastoma cells in both *in vitro* monolayer cultures and 3D spheroid models. The 3D spheroids were used the representative model since they mimic the microenvironment, characteristics, and morphology of solid tumors.

According to reviewer’s suggestion, we have changed the title to be “Combining CD3/GD2 bispecific T cell engager with human Vγ9Vδ2 T cells facilitates neuroblastoma cell targeting and killing *in vitro*.” Additionally, we have included this limitation in discussion.

“Although our study demonstrated enhanced cytotoxicity in CD3/GD2 BiTE combined with Vγ9Vδ2 T cells on 2D and 3D cell cultures, the efficacy and safety of this therapeutic combination need to be further validated in *in vivo* models and clinical trials.” Page 24 lines 556-559.

3. The data should be presented as mean +/- standard deviation or better yet, show all of the data points.

Reply: In the revised manuscript, all data is presented, with mean ± SD. Thank you for your suggestion.

4. The gdT cell expansion data should be included. For example, starting cell numbers, final cell numbers, fold-expansion, purity, immunophenotyping.

Reply: In the revised manuscript, we have included the methods and results of characterization of human Vγ9Vδ2 T lymphocytes by multiparametric immunophenotyping. Highlighted in Page 9 lines 175-184 and Page 18 lines 392-402.

5. It should be clarified whether or not only one donor was used for gdT expansion. Since donor variability is a major factor in gdT expansion and cytotoxicity, the study would benefit from including gdT cell products from multiple donors.

Reply: Thank you for your suggestion. In the revised manuscript, we have included the methods and results of characterization of human Vγ9Vδ2 T lymphocytes. As shown in Fig 4A, the activated Vγ9Vδ2 T lymphocytes were expanded from different donors. The data of cell number, percentage of Vγ9Vδ2 T cells, fold-expansion and percentage of purity were plotted from three different donors.

6. There are a few grammatical error throughout the manuscript that need to be corrected.

Reply: In the revised manuscript, we have carefully corrected all grammatical errors.

**Reviewer #3:**

Reviewer #3: Immunotherapy with BiTEs and γδT cells are both emerging approaches to cancer therapy, and have shown surprising results when used separately. In this paper, for neuroblastoma (NB), the researchers explored an innovative cancer immunotherapy approach, a bispecific T cell engager (BiTE) against CD3 and GD2, called CD3/GD2 BiTEs, which showed promising results in vitro trials. The results showed that CD3/GD2 BiTEs could effectively bind the target antigen on Jurkat and SH-SY5Y cells in 2D and 3D cell cultures and significantly enhance the cytotoxicity of Vγ9Vδ2 T cells in vitro. This combination therapy could serve as a potential alternative strategy for neuroblastoma treatment, providing new therapeutic ideas for patients with other types of solid tumors.
My additional comments are as follows:

1. No in vivo trials were conducted for CD3/GD2 BiTEs, and the lack of vivo data is a key limitation of this study, which hinders the understanding of the therapeutic potential and safety of CD3/GD2 BiTEs in living organisms. Success in vitro does not always translate to effectiveness in vivo, and due to the lack of animal testing, it is impossible to predict potential toxicity or adverse reactions in complex biological systems.

Reply: We agree with the reviewer that *in vivo* cytotoxicity would be performed to confirm the ability and safety of CD3/GD2 BiTE. CD3/GD2 BiTE only recognized the CD3 molecule but did not activate T cells or mediate cytotoxicity. However, CD3/GD2 BiTE improved the tumor specificity and avidity of Vγ9Vδ2 T cells against neuroblastoma cells. In this study, we proposed the concept for enhancing neuroblastoma cell killing by priming the activated effector cells with the binding antibody. We test the ability of CD3/GD2 BiTE to effectively redirected Vγ9Vδ2 T cells against neuroblastoma cells in both *in vitro* monolayer cultures and 3D spheroid models. The 3D spheroids were used the representative model since they mimic the microenvironment, characteristics, and morphology of solid tumors.

According to reviewer’s suggestion, we have changed the title to be “Combining CD3/GD2 bispecific T cell engager with human Vγ9Vδ2 T cells facilitates neuroblastoma cell targeting and killing *in vitro*.” Additionally, we have included this limitation in discussion.

“Although our study demonstrated enhanced cytotoxicity in CD3/GD2 BiTE combined with Vγ9Vδ2 T cells on 2D and 3D cell cultures, the efficacy and safety of this therapeutic combination need to be further validated in *in vivo* models and clinical trials.” Page 24 lines 556-559.

2. Neuroblastoma has significant genetic and phenotypic heterogeneity, but only SH-SY5Y cells were studied in vitro. Therefore, the efficacy of CD3/GD2 BiTEs on different neuroblastoma subtypes or heterogeneous tumor microenvironments is unclear. This limits the prevalence and effectiveness of CD3/GD2 BiTEs in patients with neuroblastoma.

Reply: Thank you very for your suggestion. In the revised manuscript, we have included the GD2-negative cell line, SK-N-SH cells, and CD3-negative cell line, Raji cells, for evaluating the binding specificity of CD3/GD2 BiTE by flow cytometry. As shown in Fig 3, CD3/GD2 BiTE exhibited binding reactivity to GD2-positive SH-SY5Y cells and CD3-positive Jurkat cells in dose-dependent manner. Whereas the percentage of positive cells for GD2-negative SK-N-SH cells and CD3-negative Raji cells found below 10% at all tested concentrations of CD3/GD2 BiTE. This indicated the specificity of CD3/GD3 BiTE to its target antigens. (Page 17 lines 378-380)

According to the negative binding activity of CD3/GD2 BiTE on GD2-negative SK-N-SH cells, we did not perform cytotoxicity against GD2-negative SK-N-SH cells. In this experiment, we selected GD2-positive SH-SY5y cells to be the tumor target cells since SH-SY5Y demonstrates high level expression of GD2 molecule on the cell surface. Activated Vγ9Vδ2 T cell alone without CD3/GD2 BiTE treatment and mock control using irrelevant protein were used to ensure the ability of CD3/GD2 BiTE to target neuroblastoma cell.

3. The question of why Vγ9Vδ2 T cells were selected and their advantages over other T cell subpopulations remains to be further discussed. The ability of activated γδT cells to kill a variety of solid tumors and lymphoma cells, including neuroblastoma, is mentioned in the discussion, but specific treatment cases or data should be provided to support this, especially for NB.

Reply: Thank you for your kind suggestion. We have added references to support that activated γδ T cells have been used for killing several solid tumors, particularly neuroblastoma. However, in this study, we first reported that the combination of CD3/GD2 BiTE and activated Vγ9Vδ2 T cells significantly enhances the killing of neuroblastoma cells.

“Activated γδ T cells inherently have the ability to kill a broad spectrum of solid tumors and hematological malignancies, including neuroblastoma” Page 23 lines 532-533.

4. Although this study shows that CD3/GD2 BiTEs can enhance the cytotoxicity of Vγ9Vδ2 T cells, the mechanism by which CD3/GD2 BiTEs regulates the activity of Vγ9Vδ2 T cells is unclear and can be discussed appropriately.

Reply: we described the cytotoxic mechanism of combined CD3/GD2 BiTE and activated Vγ9Vδ2 T cells against neuroblastoma cells in the discussion section and Fig 6.

“The cytotoxic mechanism of combined CD3/GD2 BiTE and activate Vγ9Vδ2 T cells against neuroblastoma cells was illustrated in Fig 6. Initially, activated Vγ9Vδ2 T cells are primed with CD3/GD2 BiTE. The scFv CD3 domain binds to CD3 molecule on Vγ9Vδ2 T cells whereas scFv-GD2 domain is free to bind GD2 molecule on neuroblastoma cells. CD3/GD2 BiTE-primed activated Vγ9Vδ2 T cells which has been improved the binding specificity and avidity against GD2 molecule are capable of targeting neuroblastoma cells. Cytotoxic granules from activated Vγ9Vδ2 T cells are released and induced apoptosis in neuroblastoma cells.” Page 25 lines 567-574.

5. CD3/GD2 BiTEs still retains mouse scFv-GD2. Although its immunogenicity and related reactions triggering HAMA have been discussed, there is still a lack of specific in vivo experimental data verification, which cannot be regarded as a conclusion.

Reply: Thank you for kindly recommending. We have removed this paragraph from the discussion section.

6. Abstract content is not clear and concise, so the purpose, methods, results and conclusions of the abstract can be more clearly divided to improve the readability of the abstract.

Reply: Abstract has been re-written according to reviewer’s recommendation. Page 2 lines 1-25 and Page 3 lines 26-35.

7. The richness and aesthetics of the picture content need to be improved, such as adding the action mechanism diagram of CD3/GD2 BiTEs.

Reply: All figures provided in the revised manuscript have been improved as recommended. The schematic illustration of cytotoxic mechanism of combined CD3/GD2 BiTE and activated Vγ9Vδ2 T cells against neuroblastoma cells was provided in Fig 6.

8. The citation format of the references is not consistent, some references do not have DOI numbers, etc. Please ensure that the format of all references conforms to the journal guidelines, and some references may need to be adjusted in terms of punctuation, italics or capitalization.

Reply: The reference format has been carefully corrected.

9. There are errors and omissions in some content formats, such as line 210:
   %% Dead tumor cells=["dead tatumor cells" (CSFE+ 7-AAD+)/"total tumor cells" (CFE+ 7AAD-) ]×100

Reply: Errors through the revised manuscript have been carefully correct.
