# Supplementary material for: Combining CD3/GD2 bispecific T cell engager with human Vγ9Vδ2 T cells facilitates neuroblastoma cell targeting and killing in vitro
Source: PLoS One. 2025 Jun 9;20(6):e0325389. doi: 10.1371/journal.pone.0325389 (PMC12148185; doi:10.1371/journal.pone.0325389)
Supplement: Attachment — Submitted filename: Response to Reviewers_PONE-D24-27594R1.docx [file pone.0325389.s005.docx]

Department of Pediatrics, Faculty of Medicine Ramathibodi Hospital,

Mahidol University, Bangkok 10400, Thailand

_____________________________________________________________________________________

March 25, 2025

Manuscript Number: **PONE-D-24-27594R1**

Title: **Combining CD3/GD2 bispecific T cell engager with human Vγ9Vδ2 T cells facilitates**

**neuroblastoma cell targeting and killing in vitro”**

**Dear reviewers,**

We would like to send a point-by-point response to the reviewer’s comments.

In the revised manuscript, we have addressed the concern of the reviewers. Changes to the text are highlighted in yellow.

We do hope that the second revised version of our study is now understandable and suitable to publish in PLOS ONE.

Thank you for your consideration of our revised manuscript.

Yours sincerely,

Suradej Hongeng, MD

Reviewer #4: Below are the main questions and suggestions.

1. The main flaw of the article is the lack of necessary in vivo experiments to verify the anti-tumor efficacy of CD3/GD2 bispecific T cell engager because in vitro experiments often fail to achieve consistent results in animal models due to overly simple systems.

Reply: We agree with the reviewer that *in vivo* cytotoxicity assay will demonstrate the accurate and precise anti-tumor efficacy of CD3/GD BiTE on neuroblastoma. In this study, we only performed *in vitro* cytotoxicity assays to propose the proof-of-concept that combining CD3/GD2 BITE with the activated Vγ9Vδ2 T cells significantly improved the neuroblastoma cell killing. The combination of our CD3/GD2 BiTE with activated Vγ9Vδ2 T cells could enhance the binding specificity and avidity of activated Vγ9Vδ2 T cells which facilitated neuroblastoma cell death *in vitro*. In this study, the combination approach required fewer effector cells 5-10 times compared with using activated Vγ9Vδ2 T cells alone. We have stated the limitations on *in vivo* anti-tumor verification on the revised manuscript (Page 24 lines 557-559).

Our title clearly stated that the concept of combining CD3/GD2 BiTE with human activated Vγ9Vδ2 T cells was verified against neuroblastoma only *in vitro*.

2. As the author mentioned, this bispecific antibody was composed of human scFv CD3 and mouse scFv GD2. Where does the sequence of scFv GD2 come from? If affinity screening and humanization modification can be made, it will be of great significance for future clinical applications.

Reply: The sequence of mouse scFv GD2 was obtained from Professor Malcolm K. Brenner, Baylor college of Medicine, Texas [1, 2]. This mouse scFv GD2 originated from the well-known GD2-specific murine monoclonal antibodies, 14G2a [3]. The monoclonal anti-GD2 14G2a has been engineered and modified to be chimeric and humanized antibodies. The anti-tumor efficacy of these antibodies them self and their combinations with cytokines, toxins or drugs have been evaluated against neuroblastoma in vitro and clinical trials. The current effector cells used are αβ T cells, γδ T cells, NK cells, NKT cells, and macrophages [4].

3. In Fig 3, the author verified the specific binding between CD3/GD2 BiTE and CD3 positive Jurkat cells. How about primary expanded Vγ9Vδ2 T cells in PBMC? Is it only a specific binding between CD3/GD2 BiTE and Vγ9Vδ2 T cells, or will it transmit activation signals, leading to activation and proliferation of Vγ9Vδ2 T cells? Besides activation and proliferation, activated Vγ9Vδ2 T cells also secrete effector cytokines such as IFN-γ. All of these are very important to prove the anti-tumor functions of CD3/GD2 BiTE.

Reply: In this study, we initially isolated human scFv against CD3 epsilon by phage panning technology. Human scFv CD3 clone 18 demonstrated the highest binding activity against CD3 epsilon by indirect ELISA. Then human scFv CD3 clone 18 was selected to construct a bispecific T cell engager with mouse scFv GD2, CD3/GD2 BiTE. As shown in Figure 3, CD3/GD2 BiTE exhibited the specific reactivity to CD3 on Jurkat cells and GD2 on SH-SY5Y cells. The result of *in vitro* cytotoxicity assay using PBMC as the effector cells showed that CD3/GD2 BiTE only recognized the CD3 molecule but did not activate T cells or mediate cytotoxicity against neuroblastoma cell lines (S1 Fig).

The suitable effector cells for CD3/GD2 BiTE should contain their own cytotoxic function. We selected human activated Vγ9Vδ2 T cells as the effector cells to combine with CD3/GD2 BiTE. CD3 epsilon is the one important subunit of CD3 complex found in both αβ T cells and γδ T cells [5]. In our study, the immunophenotyping assay of activated Vγ9Vδ2 T cells exhibited 97.1% of CD3-positive cells using APC-conjugated ant-CD3 mAb clone OTK3 (Fig 4A). The anti-CD3 mAb OKT3 binds to CD3 epsilon unit of human CD3 complex [6]. Therefore, CD3/GD2 BiTE would also bind to activated Vγ9Vδ2 T cells *via* CD3 epsilon.

Even though, CD3/GD2 BiTE only recognized the CD3 molecule but did not activate T cells or mediate cytotoxicity. The combination of activated Vγ9Vδ2 T cells and CD3/GD2 BiTE demonstrated significant *in vitro* cytotoxicity against neuroblastoma cells at 180 nM (39.90±4.09%) and 360 nM (43.57±10.46%) (Fig 4C). The dead tumor cells which are the endpoint of a cytotoxicity assay were stained with 7-AAD and measured by flow cytometer. Typically, the concentration of BiTE is required at only picomolar (pM) or micromolar (µM) level since BiTE itself can bind and activate the effector cells leading to tumor cytotoxicity [7-9]. Unlike other BiTE, the concentration of CD3/GD2 BiTE used in *in vitro* cytotoxicity assay was nanomolar level which were 10 to 20 times higher than that of other BiTEs.

According to the percentage of dead tumor cells less than 50% while using CD3/GD2 BiTE concentration at nM level in *in vitro* cytotoxicity assay, CD3/GD2 BiTE itself could not activated Vγ9Vδ2 T cells. Our CD3/GD2 BiTE could only bind to activated Vγ9Vδ2 T cells to the enhancement neuroblastoma cell targeting.

4. Functional verification is the most important part of the article. But the entire article only used one GD2 positive cell line SH-SY5Y. Other more target cells with GD2 expression should be used to repeatedly validate cytotoxic activity results. Gran B and perforin that are key molecules in cytotoxicity must be analyzed.

Reply: As mentioned earlier, mouse scFv GD2 used for CD3/GD2 BiTE construct originated from the well-known GD2-specific antibodies, 14G2a. This antibody has been engineered to be scFv and evaluated the anti-tumor efficacy, especially CAR T-cells, with several types of GD2 positive tumor cells [10]. The CD3/GD2 BiTE demonstrated the specific binding reactivity against GD2-positive SH-SY5Y cells but did not react with GD2-negative SK-N-SH cells (Fig 3). Therefore, we selected SH-SY5Y cells which exhibit a robust expression of GD2 molecule to perform *in vitro* cytotoxicity. The dead tumor cells which are the endpoint of a cytotoxicity assay were stained with 7-AAD and measured by flow cytometer.

5. We all know that CD3 is not unique to Vγ9Vδ2 T cells, so can CD3/GD2 BiTE also promote the anti-tumor function of αβT cells in vitro? Suggest the author to conduct comparative experiments to illustrate the problem.

Reply: We already performed the *in vitro* cytotoxicity of CD3/GD2 BiTE using PBMC as the effector cells. Most PBMC are αβ T cells. As shown in S1 Figure, CD3/GD2 BiTE could not promote T cell activation and cytotoxicity medication against neuroblastoma when using PBMC as the effector cells. As a consequence, we selected activated Vγ9Vδ2 T cells to be effector for combining with CD3/GD2 BiTE in this study.

6. All the figures are not well qualified and need to be improved.

Reply: All figures have been improved by reduced size and resolution to be approximately 10 MB as recommended by the journal.

Reviewer #5: Ref: PONE-D-24-27594R1

Title: Combining CD3/GD2 bispecific T cell engager with human Vγ9Vδ2 T cells facilitates

neuroblastoma cell targeting and killing in vitro

Reviewers' comments:

The authors have made detailed modifications and corrections in response all comments, addressing most issues adequately. However, there remain some areas of concern:

1. The most important deficiency of the study is lacking verifications in vivo.

Reply: We agree with the reviewer that *in vivo* cytotoxicity assay will demonstrate the accurate and precise anti-tumor efficacy of CD3/GD BiTE on neuroblastoma. In this study, we only performed *in vitro* cytotoxicity assays to propose the proof-of-concept that combining CD3/GD2 BITE with the activated Vγ9Vδ2 T cells significantly improved the neuroblastoma cell killing. The combination of our CD3/GD2 BiTE with activated Vγ9Vδ2 T cells could enhance the binding specificity and avidity of activated Vγ9Vδ2 T cells which facilitated neuroblastoma cell death *in vitro*. In this study, the combination approach required fewer effector cell numbers 5-10 times compared with using activated Vγ9Vδ2 T cells alone. We have stated the limitations on *in vivo* anti-tumor verification on the revised manuscript (Page 24 lines 557-559).

Our title clearly stated that the concept of combining CD3/GD2 BiTE with human activated Vγ9Vδ2 T cells was verified against neuroblastoma only *in vitro*.

2. No evaluation on the effectiveness across a broader range of neuroblastoma subtypes.

Reply: The sequence of mouse scFv GD2 was obtained from Professor Malcolm K. Brenner, Baylor college of Medicine, Texas [1, 2]. Mouse scFv GD2 used for CD3/GD2 BiTE construct originated from the well-known GD2-specific antibodies, 14G2a. This antibody has been engineered to be scFv and evaluated the anti-tumor efficacy, especially CAR T-cells, with several types of GD2 positive tumor cells [10]. The CD3/GD2 BiTE demonstrated the specific binding reactivity against GD2-positive SH-SY5Y cells but did not react with GD2-negative SK-N-SH cells (Fig 3). Therefore, we selected SH-SY5Y cells which exhibit a robust expression of GD2 molecule to perform *in vitro* cytotoxicity.

3. Further discussion on the rationale for choosing Vγ9Vδ2 T cells is needed.

Reply: We have already discussed the rationale of choosing the expanded Vγ9Vδ2 T cells in discussion section. (Page 23 lines 528-533)

“The adoptive transfer of expanded Vγ9Vδ2 T cells has been shown to be safe and well tolerated in clinical trials. γδ T cell-based immunotherapy, especially Vγ9Vδ2 T cells, holds significant promise for tumor treatment due to low toxicity profile and lack of dependency on an individual’s major histocompatibility complex, making therapeutic γδ T cells from healthy donors a viable option to reduce on-target off-tumor effects [43].”

As recommended by reviewer, we have added the rationale of choosing allogeneic γδ T cells to combine with CD3/GD2 BiTE in discussion section. (Page 24-25 lines 560-566)

“Allogenic γδ T cells provide several advantages in cancer immunotherapy which are MHC-independent tumor recognition and reduced risk of graft-versus-host disease (GVHD [11, 12]. In addition, the mass production of “off-the-shelf” allogeneic γδ T cells can be produced since γδ T cells can be expanded, manufactured, and stored which are readily available for use in multiple patients [13, 14]. Therefore, allogeneic γδ T cells derived from a heathy donor could be considered to combine with CD3/GD2 BiTE for further verification the anti-tumor efficacy in animal models and clinical trials.”

**References**

1.	Yvon E, Del Vecchio M, Savoldo B, Hoyos V, Dutour A, Anichini A, et al. Immunotherapy of Metastatic Melanoma Using Genetically Engineered GD2-Specific T cells. Clin Cancer Res. 2009;15(18): 5852-60. <https://doi.org/10.1158/1078-0432.CCR-08-3163>

2.	Louis CU, Savoldo B, Dotti G, Pule M, Yvon E, Myers GD, et al. Antitumor activity and long-term fate of chimeric antigen receptor–positive T cells in patients with neuroblastoma. Blood. 2011;118(23): 6050-6. <https://doi.org/10.1182/blood-2011-05-354449>

3.	Mujoo K, Kipps TJ, Yang HM, Cheresh DA, Wargalla U, Sander DJ, et al. Functional properties and effect on growth suppression of human neuroblastoma tumors by isotype switch variants of monoclonal antiganglioside GD2 antibody 14.18. Cancer Res. 1989;49(11): 2857-61. PMID: 2720646.

4.	Philippova J, Shevchenko J, Sennikov S. GD2-targeting therapy: a comparative analysis of approaches and promising directions. Front Immunol. 2024;15: 1371345. <https://doi.org/10.3389/fimmu.2024.1371345> PMID: 38558810.

5.	Morath A, Schamel WW. αβ and γδ T cell receptors: Similar but different. J Leukoc Biol. 2020;107(6): 1045-55. <https://doi.org/10.1002/JLB.2MR1219-233R> PMID: 31994778.

6.	Kjer-Nielsen L, Dunstone MA, Kostenko L, Ely LK, Beddoe T, Mifsud NA, et al. Crystal structure of the human T cell receptor CD3 epsilon gamma heterodimer complexed to the therapeutic mAb OKT3. Proc Natl Acad Sci USA. 2004;101(20): 7675-80. <https://doi.org/10.1073/pnas.0402295101> PMID: 15136729.

7.	Yankelevich M, Kondadasula SV, Thakur A, Buck S, Cheung NK, Lum LG. Anti-CD3 × anti-GD2 bispecific antibody redirects T-cell cytolytic activity to neuroblastoma targets. Pediatr Blood Cancer. 2012;59(7): 1198-205. <https://doi.org/10.21203/rs.3.rs-3570311/v1> PMID: 22707078.

8.	Zeng X, Zhang H, Guo J, Yang D, Zhu Y, Liu N, et al. A novel bispecific T-cell engager using the ligand-target csGRP78 against acute myeloid leukemia. Cell Mol Life Sci. 2024;81(1): 371. <https://doi.org/10.1007/s00018-024-05410-0> PMID: 39196413.

9.	Ganesan R, Chennupati V, Ramachandran B, Hansen MR, Singh S, Grewal IS. Selective recruitment of γδ T cells by a bispecific antibody for the treatment of acute myeloid leukemia. Leukemia. 2021;35(8): 2274-84. <https://doi.org/10.1038/s41375-021-01122-7> PMID: 33526858.

10.	Craddock JA, Lu A, Bear A, Pule M, Brenner MK, Rooney CM, et al. Enhanced tumor trafficking of GD2 chimeric antigen receptor T cells by expression of the chemokine receptor CCR2b. J Immunother. 2010;33(8): 780-8. <https://doi.org/10.1097/CJI.0b013e3181ee6675> PMID: 20842059.

11.	Sebestyen Z, Prinz I, Déchanet-Merville J, Silva-Santos B, Kuball J. Translating gammadelta (γδ) T cells and their receptors into cancer cell therapies. Nat Rev Drug Discov. 2020;19(3):169-84. <https://doi.org/10.1038/s41573-019-0038-z>

12.	Hu Y, Hu Q, Li Y, Lu L, Xiang Z, Yin Z, et al. γδ T cells: origin and fate, subsets, diseases and immunotherapy. STTT. 2023;8(1):434. <https://doi.org/10.1038/s41392-023-01653-8>

13.	Makkouk A, Yang XC, Barca T, Lucas A, Turkoz M, Wong JTS, et al. Off-the-shelf Vδ1 gamma delta T cells engineered with glypican-3 (GPC-3)-specific chimeric antigen receptor (CAR) and soluble IL-15 display robust antitumor efficacy against hepatocellular carcinoma. J Immunother Cancer. 2021;9(12). <https://doi.org/10.1136/jitc-2021-003441> PMID: 34916256.

14.	Jhita N, Raikar SS. Allogeneic gamma delta T cells as adoptive cellular therapy for hematologic malignancies. Explor Immunol. 2022;2(3): 334-50. <https://doi.org/10.37349/ei.2022.00054> PubMed PMID: 35783107.
