# Supplementary material for: Combining CD3/GD2 bispecific T cell engager with human Vγ9Vδ2 T cells facilitates neuroblastoma cell targeting and killing in vitro
Source: PLoS One. 2025 Jun 9;20(6):e0325389. doi: 10.1371/journal.pone.0325389 (PMC12148185; doi:10.1371/journal.pone.0325389)
Supplement: Attachment — Submitted filename: Response to Reviewer PONE-D24-27594R2.docx [file pone.0325389.s006.docx]

Department of Pediatrics, Faculty of Medicine Ramathibodi Hospital,

Mahidol University, Bangkok 10400, Thailand

_____________________________________________________________________________________

April 25, 2025

Manuscript Number: **PONE-D-24-27594R2**

Title: **Combining CD3/GD2 bispecific T cell engager with human Vγ9Vδ2 T cells facilitates**

**neuroblastoma cell targeting and killing in vitro”**

**Dear reviewer,**

We would like to thank you for your valuable comments and suggestions. We have addressed your concerns and here are the responses regarding your comments.

We do hope that this revised version of our study is suitable to publish in PLOS ONE.

Thank you for your consideration of our revised manuscript.

Yours sincerely,

Suradej Hongeng, MD

Reviewer #4: The author addressed that CD3/GD2 BiTE only recognized the CD3 molecule but did not activate T cells. I still insist that an effective anti-tumor BiTE should be able to activate anti-tumor effector cells in vivo, not just as a target. If an anti-CD3 scFv sequence that can both target and activate CD3+ T cells could be selected during screening, it will have a more promising application prospect.
In addition, the author conducted experiments using PBMCs and did not observe an increase in anti-tumor cytotoxicity. So what about activated T cells using CD3/CD28 beads? If the author emphasizes that their BiTE can only bind to activated V γ 9V δ 2 T cells and enhance the targeting to GD2+ tumor cells, a scFv targeting anti-V γ 9V δ 2 TCR should be screened instead of targeting CD3 molecules.
Since the anti-tumor efficacy in vivo cannot be achieved, it is necessary to verify using several different target cells and effector cells in vitro. However, I did not see the authors make every effort to improve it in the revised manuscript.

**Reply**: We totally agree with reviewer that an effective BiTE should not only bind but also activate T cell function and mediate cytotoxic killing both *in vitro* and *in vivo*. Our first attempt, in this study, is to screen for effective human scFv CD3 that could activate T cell function and mediate cytotoxicity. Unfortunately, we obtained human scFv-CD3 clone 18 that could only bind to CD3 molecules. However, binding human scFv CD3 clone 18 could be used for constructing CD3/GD2 BiTE by joining with mouse scFv GD2 via flexible linker. The CD3/GD2 BiTE could be alternatively used for enhancing the binding specificity and avidity of the effector cells, activated Vγ9Vδ2 T cells, to facilitate neuroblastoma cell killing *in vitro*. The CD3/GD2 BiTE and activated Vγ9Vδ2 T cells combination approach required fewer effector cells 5-10 times compared with using activated Vγ9Vδ2 T cells alone.

After conducting *in vitro* cytotoxicity assay using PBMC as the effector cells, we found that CD3/GD2 BiTE could not promote T cell activation and cytotoxicity mediation against neuroblastoma. We also thought that activated T cells might be used as an effector cell instead. However, literature reported that T cell receptor-CD3 (TCR-CD3) complex is internalized into T cells upon ligation with MHC:peptide complexes resulting in downmodulation of TCR-D3 cell surface on activated T cells [1, 2]. Therefore, we investigated the expression of CD3 molecules on activated T cells by flow cytometry assay. The activated T cells were prepared by incubating PBMC with 2 µg/ml anti-CD3 (clone OKT3) (BioLegend, USA), 5 µg/ml anti-CD28 (clone 28.2) (BioLegend, USA) and 30 U/ml IL-2 at 37°C in 5% CO_2_ for 2 days. Then activated T cells were performed cell surface and intracellular staining using FITC conjugated anti-human CD3 antibody (clone UCHT1) (BioLegend, USA). As shown in the table below, the percentage of CD3 positive cells from cell surface staining was only 2.41% while intracellular staining demonstrated CD3-positive cells of 61.9%. This indicated that CD3 molecules were internalized into T cells after *in vitro* T cell activation. Therefore, activated T cells were not suitable to use as an effector cell for combining with CD3/GD2 BiTE.

| Cell type | %CD3 positive cells | | | |
| --- | --- | --- | --- | --- |
|  | Unstained | Cell surface staining | Unstained | Intracellular staining |
| PBMC | 0 | 74.3 | ND | ND |
| Activated T cells | 0.03 | 2.41 | 0.08 | 61.9 |

As mentioned earlier, activated Vγ9Vδ2 T cells were used as the appropriate effector cells to combine with CD3/GD2 BiTE since they can promote the cytotoxicity themselves. In addition, the immunophenotype of activated Vγ9Vδ2 T cells revealed 97.1% of CD3-positive cells using APC-conjugated ant-CD3 mAb clone OTK3 (Fig 4A). This indicated that, after expansion, the CD3 molecules still located on the surface of activated Vγ9Vδ2 T cells. According to the surface expression of CD3 molecules and cytotoxic function ability, the activated Vγ9Vδ2 T cells could be used for combining with CD3/GD2 BiTE to enhance neuroblastoma targeting and killing.

In this manuscript, all reviewers have provided valuable time to give us comments and suggestions. Your comments and suggestions are appreciated and have been carefully considered. Compared to the first version of this manuscript, the latest one was markedly improved by peer-review process. We would like to thank you very much.

**References**

1.	Liu H, Rhodes M, Wiest DL, Vignali DA. On the dynamics of TCR:CD3 complex cell surface expression and downmodulation. Immunity. 2000;13(5): 665-75. <https://doi.org/10.1016/S1074-7613(00)00066-2> PMID: 11114379.

2.	van der Donk LEH, Ates LS, van der Spek J, Tukker LM, Geijtenbeek TBH, van Heijst JWJ. Separate signaling events control TCR downregulation and T cell activation in primary human T cells. Immun Inflamm Dis. 2021;9(1): 223-38. <https://doi.org/10.1002/iid3.383> PMID: 33350598.
